# Outcomes of protease inhibitor-based antiretroviral therapy amongst children and associated-factors in Yaoundé, Cameroon

**DOI:** 10.1371/journal.pone.0213900

**Published:** 2019-03-18

**Authors:** Anne Esther Njom Nlend, Cathya Ornella Guessong, Annie Carole Nga Motaze, Claudian Soffo, Paul Olivier Koki Ndombo, Lionel Tsambang, Joseph Fokam

**Affiliations:** 1 Essos Hospital Centre, National Insurance Fund, Yaoundé, Cameroon; 2 Higher Institute of Medical Technology Nkolondom, Department of Clinical Sciences, University of Douala, Cameroon; 3 Cameroon Association for Support of Persons and families affected by AIDS, Yaoundé, Cameroon; 4 Faculty of Medicine and Biomedical Sciences, University of Yaoundé 1, Yaoundé, Cameroon; 5 Mother-Child Centre, Chantal BIYA Foundation, Yaoundé, Cameroon; 6 National HIV drug resistance prevention and surveillance working group, Ministry of Public Health, Yaoundé, Cameroon; 7 Virology Laboratory, Chantal BIYA International Reference Centre for Research on HIV/AIDS prevention and management, Yaoundé, Cameroon; Universita degli Studi di Roma Tor Vergata, ITALY

## Abstract

**Background:**

There are limited data on protease inhibitor (PI)-based antiretroviral therapy (ART) amongst children in resource-limited settings, for informing on optimal paediatric regimens.

**Objective:**

To evaluate therapeutic response to PI-based ART amongst HIV-infected Cameroonian children.

**Methods:**

A retrospective study was conducted amongst children aged 2–18 years receiving a PI-based ART at the Essos Hospital Centre (EHC), Yaounde, Cameroon. Primary end points were therapeutic success on PI-based ART, defined as clinical success (WHO I/II clinical stage), immunological success (CD4 ≥ 500/mm^3^) and viral suppression (viral load [VL]<1000 copies/ml). Factors associated with therapeutic success were assessed in uni- and multivariate analysis using SPSS software v.2.0; with p<0.05 considered statistically significant.

**Results:**

A total of 71 eligible children on PI-based ART were enrolled (42 on initial and 29 on substituted regimens), with a median age of 8 [IQR: 5–12] years and mean duration on ART of 7 years. Following therapeutic responses, all (100%) experienced clinical success, 95.2% experienced immunological success (91.7% on initial and 97.2% on substituted PI/r-based regimens) and 74.7% viral suppression. In univariate analysis, viral suppression was associated with: younger age (p<0.0001), living with parents as opposed to guardians (p = 0.049), and the educational level (p<0.0001). In multivariate analysis, only the age ranges of 10–14 years (OR: 0.22 [0.07–0.73]) and 15–18 years (OR: 0.08 [0.02–0.57]), were determinants of poor viral suppression.

**Conclusion:**

Among these Cameroonian children, PI-based ART confers favourable clinical and immunological outcomes. The poor rate of viral suppression was mainly attributed to adolescence (10–18 years).

## Introduction

Almost 1.8 million children are living with HIV (CLHIV) worldwide, of whom 1.6 million are from sub-Saharan Africa (SSA) [1,2]. During the last decade, the increasing access of antiretroviral therapy (ART) has improved the survival rate amongst CLHIV in SSA, with about 50% paediatric ART coverage [2,3]. Progress in the therapeutic management of CLHIV in SSA has ensured the revision of eligibility criteria both) for initiating first-line and for switching to second-line ART regimens following the world health organisation (WHO) recommended public health approach [4,5]. Of note, based on recent evidence and the effective implementation of prevention of mother-to-child transmission (PMTCT) option B+ in SSA settings, current guidelines recommend ART regimens consisting of ritonavir-boosted protease-inhibitor (PI/r) as the preferred first line option in children below 3 years, and as preferred second-line option after failure to a non-nucleoside reverse transcriptase inhibitor (NNRTI)-based ART regimen [5].

In spite of the effectiveness of current ART strategy in both adult and children populations, achieving the expected target for viral suppression (i.e. 90% viral load below 1,000 copies/mL) among children is more challenging compared to adults [6]. This is particularly true in the frame of very high viral loads in paediatric populations, limited paediatric therapeutic options, the wide use of drugs with low-genetic barriers to resistance (i.e. the majority of CHIV still receiving NNRTI-based regimens) and the paucity of evidence on response to PI-based regimens either as first- or second-line ART in SSA [6,7]. As current efforts in viral monitoring of CLHIV would increase the switch to PI/r-based regimens, it becomes crucial to set-up relevant strategies for: (a) ensuring a long-term successful initial regimen, (b) ensuring viral re-suppression once on second-line regimen; and (c) understanding the local factors associated with treatment outcomes [7].

Amongst Cameroonian CLHIV, findings revealed poor therapeutic response, especially during adolescence, and high rates of acquired HIV drug resistance (HIVDR) among those failing NNRTI-based ART [8,9]. Of note, this high rate of resistance was favoured by a prolonged exposure to failing regimens, which in turn prompts the accumulation of DR mutations [10,11]. Thus, in the frame of limited knowledge about response to paediatric PI/r-containing regimens, our study objectives were to evaluate the therapeutic (clinical, immunological and virological) response of children receiving a PI/r-based ART, to compare the response on PI/r used in first- versus second-line combinations, and study the determinants of therapeutic response.

## Materials and methods

### Study design and site description

A retrospective cohort-study was conducted amongst children aged 2–18 years receiving PI/r-based regimens either as initial (i.e. first-line) or substituted (i.e. second-line) ART at the paediatric department of the Essos Hospital Centre (EHC) in Yaoundé, the capital city of Cameroon, from 2005 to 2016.

The EHC is an approved treatment centre for HIV-infection in adults, adolescents and children; and paediatric ART was launched onsite by 2005. At this study site, CLHIV on PI/r-based regimens receive at early age a syrup of ritonavir boosted with lopinavir (LPV/r), while those at older age or weighting > 10kg received pills of LPV/r. Detailed site description is provided elsewhere [8,9].

### Study participants and sampling procedure

Following an exhaustive sampling at the study site (EHC), all CLHIV fulfilling the following criteria were enrolled in the study: (a) aged 2–18 years, (b) currently receiving a PI/r-based ART regimen, (c) treated for at least 6 months with a PI/r-based regimen and (d) reported to be adherence. A study participant was excluded if: (a) placed on PI/r-based ART due to adverse events to an NNRTI-based ART or due to comorbidities, (b) transfer out of the study site, (c) lost to follow-up or dead, and (d) data unavailable.

### Data collection

Data were collected from the medical records of each eligible child monitored at the study site and the following variables were abstracted: socio-demographic data (age, gender, level of education, family/guardian); clinical data (ART regimen, duration of ART, WHO clinical staging, CD4 T lymphocyte cells count, viral load measurement at initiation of PI/r-based ART and at the last monitoring); see mini data set attached as supporting file,S1 Table.

### Data analysis

Collected data were entered into an electronic datasheet developed using Epi DATA software and analysis was done with the STATA version 3.1 and Microsoft Excel 2010. Statistical significance was set at 5%, using 95% confidence interval (CI). The predictive factors for clinical, immunological and virological conditions were identified in univariate and multivariate analysis using logistic regression at a significance threshold of 5%. Following the definition of therapeutic response, clinical success was defined as WHO stage I or II at the moment of the study; immunological success was defined as CD4 >500 cells/mm^3^; and viral suppression was defined as HIV-1 RNA <1000 RNA copies/ml.

### Ethical considerations

Ethical clearance for the study was obtained from the Institutional review board (IRB) of the Essos Hospital Centre under the reference number 2017/22/CE-CHE; the Hospital Directorate provided an administrative authorization; as per approval from the IRB on the consent procedure, a written proxy-informed consent was obtained from the respective parent/caregiver; a verbal assent was obtained from the child as he/she grew-up; and all data were processed under strict confidentiality and privacy by using unique identifiers.

## Results

### Characteristics of the study population

**Fig 1:**
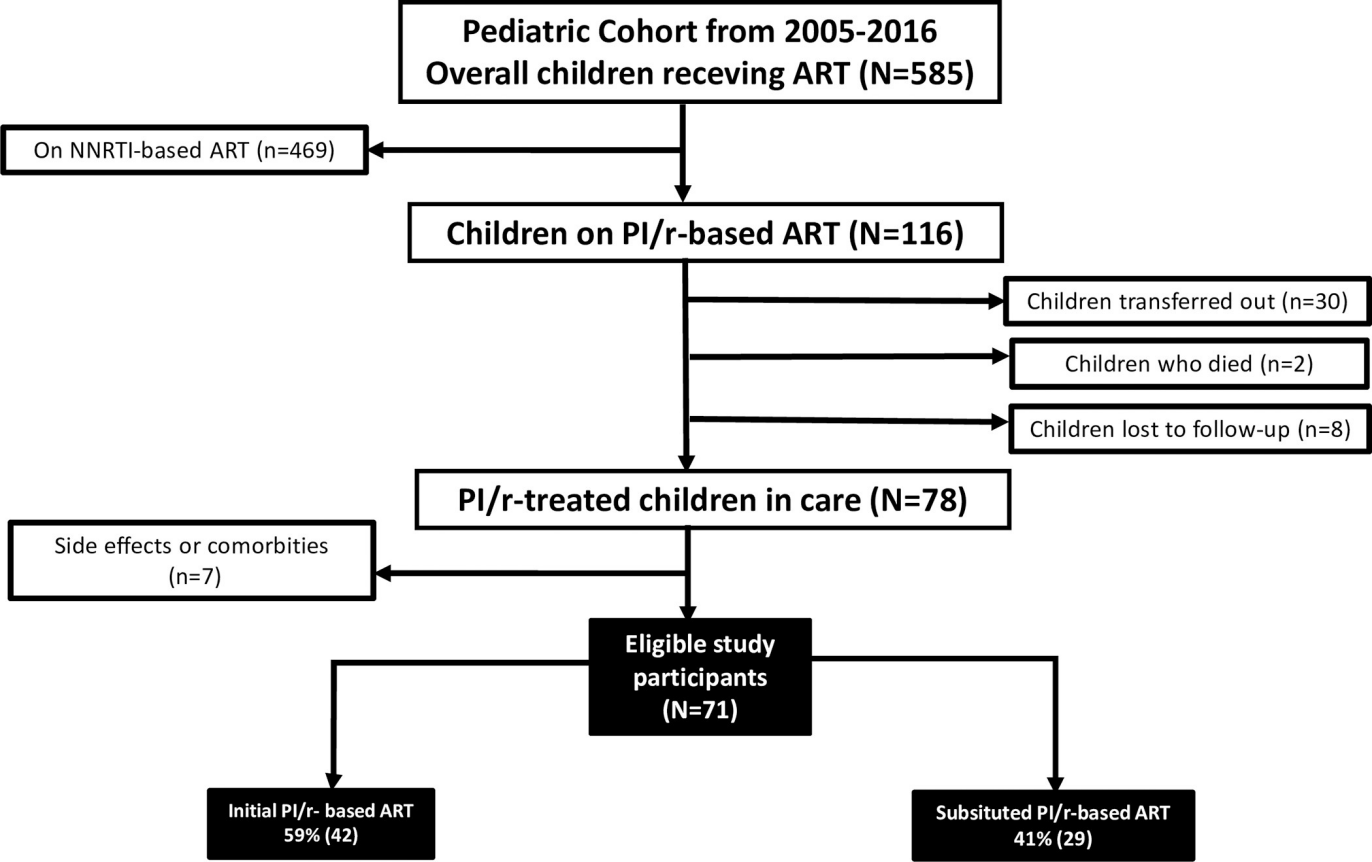
Flow chart for enrolment of the study participants.

Out of 108 CLHIV in the cohort of those receiving PI/r-based ART, 37 were excluded following documented reports of switched to PI/r-based ART due to adverse events or comorbidities, transferred out, lost to follow-up or deaths; giving a total of 71 participants eligible for analysis (Fig 1).

Of the 71 eligible participants, 59.2% were male and 2/3 of them were older than 10 years (i.e. adolescents). Up to 30% were orphans of at least one parent, with the highest proportion of orphans (41.4%) found amongst those on second-line PI/r-based ART (Table 1).

**Table 1:** Characteristics of the study population.

Sociodemographic data	Treatment regimens				Total	
	*Substitute PI based ART*		*Initial PI based ART*			
	*Number* *(n = 29)*	*Frequency* *(%)*	*Number* *(n = 42)*	*Frequency* *(%)*	*Number* *(n = 71)*	*Frequency* *(%)*
***Gender***						
*Female*	*11*	*37*.*9*	*18*	*42*.*9*	*29*	*40*.*8*
*Male*	*18*	*62*.*1*	*24*	*57*.*1*	*42*	*59*.*2*
***Age (years)***						
*2–9*	*1*	*3*.*5*	*42*	*100*	*43*	*60*.*5*
*10–14*	*19*	*65*.*5*	*0*	*0*	*19*	*26*.*8*
*15–18*	*9*	*31*	*0*	*0*	*9*	*12*.*7*
***Family status***						
*Orphan*	*12*	*41*.*4*	*10*	*23*.*8*	*22*	*39*
*Non orphan*	*17*	*58*.*6*	*32*	*76*.*2*	*49*	*61*
***Level of education***						
*None*	*0*	*0*	*9*	*21*.*4*	*9*	*12*.*7*
*Primary*	*12*	*41*.*4*	*33*	*78*.*6*	*45*	*63*.*4*
*Secondary*	*17*	*58*.*6*	*0*	*0*	*17*	*23*.*9*
***Living with parent or guardian***						
*Parent*	*20*	*69*	*37*	*88*	*57*	*80*.*3*
*Guardian*	*9*	*31*	*5*	*12*	*14*	*19*.*7*

ART: antiretroviral therapy; PI/r: ritonavir-boosted protease inhibitor.

Mean age of children at the moment of ART initiation was 7.1 months for those on first-line PI/r-based ART and 68.3 months for those on second-line PI/r-based ART. At the moment of the study, the mean age for children on a first- and second-line PI/r-based ART was 5.5 years and 13.4 years respectively. The median duration of ART was 84 months overall.

### Clinical, immunological and virological responses to ART

In the entire study population, the clinical success was observed to be 100% (71/71) in both groups. Of note, majority of children were at the WHO stage I (94.4%) while the remaining fewer cases (5.6%) were classified as WHO II. Following immunological success, 95.2% had a CD4 count greater than 500 cells/μl. following viral suppression, 74.7% had a plasma viral load below 1000 RNA copies/mL. Of note, viral suppression was slightly higher amongst children on a first-line (76.2%) versus those on second-line PI/r-based regimen (72.4%); as shown in Table 2.

**Table 2:** Therapeutic outcomes of children on protease inhibitor-based antiretroviral therapy.

Variables	Treatment regimens				Total	
	*Substitute PI based ART*		*Initial PI based treatment*			
	*Number* *(n = 29)*	*Frequency* *(%)*	*Number* *(n = 42)*	*Frequency* *(%)*	*Number* *(n = 71)*	*Frequency* *(%)*
***WHO clinical stage***						
*WHO I*	*25*	*86*.*2*	*42*	*100*	*67*	*94*.*4*
*WHO II*	*4*	*13*.*8*	*0*	*0*	*4*	*5*.*6*
*WHO III*	*0*	*0*	*0*	*0*	*0*	*0*
*WHO IV*	*0*	*0*	*0*	*0*	*0*	*0*
***Absolute CD4 cell count***						
*<350*	*1*	*4*.*2*	*0*	*0*	*1*	*1*.*7*
*350–499*	*1*	*4*.*2*	*1*	*2*.*8*	*2*	*3*.*3*
*> 500*	*22*	*91*.*6*	*35*	*97*.*2*	*57*	*95*
***Viral load (copies/ml)***						
*<1000*	*21*	*72*.*4*	*32*	*76*.*2*	*53*	*74*.*7*
*≥1000*	*8*	*27*.*6*	*10*	*23*.*8*	*18*	*25*.*3*

ART: antiretroviral therapy.

#### Factors associated to therapeutic success (univariate and multivariate analysis)

As substantial difference in response was found mainly with viral load, associated factors were then evaluated with respect to this variable. It appeared that, the child age at the moment of the study, the educational level, living with their parents/guardians, were independent factors of viral suppression (viral load <1000 RNA copies/mL). Particularly, older children were less likely to experience viral suppression. Children aged 15–18 years were nine times less likely to achieve viral suppression compared to those aged ≤10 years (Odd Ratio = 0.08); similarly, children aged 10–14 years were eight times less likely to achieve viral suppression compared to those aged ≤10 years (Odd Ratio = 0.22), as shown in Tables 3 and 4.

**Table 3:** Factors associated with viral suppression in univariate analysis.

Independent variables	Viral suppression (%)		Khi^2^ p value at 5%
	Yes	No	
***Current age of the child (years)***			*0*.000*
*2–9*	*100*	*0*	
*10–14*	*63*.*6*	*36*.*4*	
*15–18*	*25*	*75*	
***Level of education***			*0*.000*
*None*	*100*	*0*	
*Primary*	*100*	*0*	
*Secondary*	*36*.*4*	*63*.*6*	
***Line of treatment***			*0*.*719*
*Initial PI/r-based ART*	*76*.*2*	*23*.*8*	
*Second-line PI/r-based ART*	*72*.*4*	*27*.*6*	
***Living with parents or guardians***			*0*.049*
*Parent*	*87*.*9*	*12*.*1*	
*Guardian*	*57*.*1*	*42*.*9*	
***Gender***			***0*.*592***
*Male*	*81*.*5*	*18*.*5*	
*Female*	*84*.*6*	*15*.*4*	
***Orphan or not***			***0*.*631***
*Orphan*	*78*.*6*	*21*.*4*	
*Non orphan*	*84*.*6*	*15*.*4*	

ART: antiretroviral therapy; PI/r: ritonavir boosted protease inhibitor; viral suppression is defined as plasma viral load < 1000 copies/mL.

*Significant

**Table 4:** Factors associated to viral suppression in multivariate analysis.

Independent variables	P value	Odd Ratio	95% confidence Interval
*Current age of the child (years)*			
*2–9 (ref)*	*-*	*-*	*-*
*10–14*	*0*.000*	*0*.*22*	*[0*.*07 ; 0*.*73]*
*15–18*	*0*.000*	*0*.*08*	*[0*.*02 ; 0*.*57]*
*Level of education*			
*None (ref)*	*-*	*-*	*-*
*Primary*	*0*.*095*	*7*.*98*	*[0*.*73 ; 23*.*5]*
*Secondary*	*0*.*372*	*0*.*57*	*[0*.*17 ; 1*.*95]*
*Living with parents or guardians*			
*Parent (ref)*	*-*	*-*	*-*
*Guardian*	*0*.*069*	*0*.*18*	*[0*.*03 ; 1*.*14]*
*Treatment regimen*			
*Initial PI based treatment (ref)*	*-*	*-*	*-*
*Second-line PI based ART*	*0*.*648*	*0*.*55*	*[0*.*04 ; 6*.*68]*

ART: antiretroviral therapy; Ref: Reference; PI/r: ritonavir boosted protease inhibitor; viral suppression is defined as viral load < 1000 copies/mL.

*Significant

## Discussion

With paucity of data on response to paediatric PI/r-based ART and their determinants in SSA, generating evidence on clinical, immunological and virological responses would serve in designing strategies with maximal outcomes in these settings.

As primary outcomes, clinical and immunological status appear satisfactory, while viral suppression on PI/r-based ART was considered suboptimal (75%) among these children, especially regarding the duration on ART (median: about seven years). Therefore, even in SSA settings, our findings support the use of viral load in routine monitoring in order to detect ART failure on time [8]. Interestingly, the similar rate of viral suppression first and second-line PI/r-based ART-experiencing children suggest comparable risk of ART failure in both ART lines in these settings [12–15].

Based on the high rate of virological failure on PI/r-containing regimens (especially for those on second-line with a viral load above 1000 RNA copies/mL), it appears urgent to question the introduction of third-line of ART using innovative molecules such as integrase strand-transfer inhibitors in pediatric care [11]. Such new drugs might help CLHIV failing second-line PI/r-based ART to achieve viral re-suppression and regain a normal life span [16]. Our results are similar to data from Uganda (15% virological failure after 48 months) and from Thailand (81% of children with viral suppression after 48 weeks of second line ART) [13,14]. Nonetheless, our rate of virological failure on second-line PI/r-based ART appears more favourable (23.8%) compared to reports from Bangui among similar children (47% virological failure after 18–30 months) [12]. A possible reason could be in the difference in monitoring between the countries [16]. Also, resistance profile is unknown in the aforementioned studies, which in turn require further investigations [16–18].

Regarding the median time on second line ART (7 years), we could there postulate that at least 20% of our children are on a failing regimen once they are treated for a similar period of time. As second-line in paediatric remains the ultimate ART-line in our context, an appeal for access to HIVDR testing appear as a key weapon in combatting risk of multi-resistance by sequencing the most potentially active ingredients, as reported in South-African children [15]. Beside the use of innovative drugs, SSA countries should therefore consider resistance testing into routine clinical practice for children with such complex viral genotypic profile [19–21].

As viral suppression in children appears to be associated with younger age (<10 years old), living with parents, being on first-line PI/r-based ART regimens and having a low educational level, it would be of great clinical importance to design a closer or specific monitoring strategy once a child enters adolescence, for those on a long-term second-line regimen. Such corrective measures would improve adherence and sustain viral suppression [22].

Our findings would have provided stronger evidence with adequate measurements of adherence (self reported in at study site) and with HIVDR profiling to confirm ART failure or to suspect non-adherence/poor bioavailability. The threshold used for viral suppression suggests on-going replication and risk for selecting resistant variants for children with low-level viremia (viral load between 40–999 RNA copies/mL) [23–24].

Another limitation to this study is the fact that children with higher age might also reflect a longer ART exposure. Of note, in the absence of data on adherence and resistance, the poorer virological outcome observed among adolescents could not be attributed solely to a worse adherence, but also to their potential longer duration on ART as compared to younger children. This therefore calls for more emphasis on the need of viral load in monitoring ART, and timely resistance test and adherence assessment in case of virological failure.

## Conclusion

After seven years of PI/r-based ART experience, CLHIV in a typical urban setting of Cameroon have favourable clinical and immunological outcomes. However, the poor rate of viral suppression (<80%) requires interventions in the clinical settings mainly towards adolescents and those on a second-line PI-based regimen.

## Supporting information

S1 Table Mini data set of children enrolled on protease inhibitor treatment as Essos Hospital Centre. ART: antiretroviral therapy, NNRTI: non-nucleoside reverse transcriptase inhibitor; PI/r: ritonavir boosted protease inhibitor;.
(XLS)
